# Mental health problems among healthcare professionals following the workplace violence issue-mediating effect of risk perception

**DOI:** 10.3389/fpsyg.2022.971102

**Published:** 2022-08-31

**Authors:** Deping Zhong, Chengcheng Liu, Chunna Luan, Wei Li, Jiuwei Cui, Hanping Shi, Qiang Zhang

**Affiliations:** ^1^School of Social Development and Public Policy, Beijing Normal University, Beijing, China; ^2^Beijing Nutrinst Medical Research Institute, Beijing, China; ^3^Department of Oncology, The First Hospital, Jilin University, Changchun, China; ^4^Department of Gastrointestinal Surgery/Clinical Nutrition, Capital Medical University Affiliated Beijing Shijitan Hospital, Beijing, China

**Keywords:** issue salience, healthcare professionals, workplace violence (WPV), mental health, risk perception

## Abstract

Although there have been numerous studies on mental wellbeing impairment or other negative consequences of Workplace Violence (WPV) against healthcare professionals, however, the effects of WPV are not limited to those who experience WPV in person, but those who exposed to WPV information indirectly. In the aftermath of “death of Dr. Yang Wen,” a cross-sectional study was conducted to explore the psychological status of healthcare professionals. A total of 965 healthcare professionals from 32 provinces in China participated in our research. The prevalence rates of Post-Traumatic Stress Disorder (PTSD) symptoms, depression, anxiety among healthcare professional in the current study were 25.60, 46.01, and 27.88%, respectively. Moreover, our research suggested that the awareness of WPV-incident had a significant association with PTSD symptoms. In addition, risk perception was shown to mediate the effect of WPV awareness on PTSD symptoms. Furthermore, the present research also found a U-shaped relationship between issue salience and PTSD symptoms, and the relationship between issue salience and anxiety, indicating that higher awareness of WPV issue was negatively related to mental health status (including PTSD and anxiety) but only to the points at which there were no additional effects of more issue salience. This study highlighted that more protective measures for healthcare professionals need to be implemented in response to potential WPV events. More importantly, risk perception was found to mediate the effect of WPV issue salience on PTSD symptoms, it is critical to reduce the mental health burden through intervening in risk perception.

## Introduction

The overwhelming spread of COVID-19 appended an additional burden on the already stressful medical environment (Said and El-Shafei, 2021), which has triggered a wave of workplace violence (WPV) against healthcare professionals due to low healthcare professional-patient ratio (Xie et al., 2021), limited communication between health professionals and patients (Liu R. et al., 2021). According to the International Committee of the Red Cross report, more than 600 incidents of WPV were targeted at healthcare professionals, medical infrastructure, etc., across 40 countries in Asia, Americas, Africa, and the Near and Middle East regions during the first 6 months of COVID-19 pandemic, with much more remaining unnoticed (Ghareeb et al., 2021). However, WPV is not just the product of COVID-19 pandemic, healthcare professionals had been at an elevated risk for experiencing WPV before the COVID-19 pandemic. The World Health Organization reported that 8–38% of healthcare professionals suffered WPV at some point in their career (Singh et al., 2019). In China, a 2016 survey among 1,024 healthcare personnel revealed that more than 75% of healthcare personnel had experienced at least one form of WPV (Zhang et al., 2018).

According to the well-established evidences, healthcare professionals exposed to WPV were at an elevated risk of mental wellbeing impairment or other negative consequences (Sharma et al., 2019; Ghareeb et al., 2021). However, the case of “the attack on Dr. Yang Wen” highlighted that the existing studies about the effects of WPV against healthcare professionals were most likely just the “tip of the iceberg,” more studies need to explore the indirect WPV exposure, which refers to the awareness of some direct WPV victims (which is also known as issue salience), in other healthcare professionals.

On 24 December 2019, Dr. Yang Wen was brutally murdered by a relative of a patient. After this traumatizing incident, the information rapidly spread on the internet, and healthcare professionals all over the country were shocked by this brutal attack. Four days after this brutal attack upon healthcare professionals, the Standing Committee of the National People’s Congress issued the comprehensive law Basic Medical and Healthcare Promotion Act, which emphasized the importance of protecting healthcare professionals from violence. This salient WPV issue had attracted much attention from the government and from the public. In addition, this public issue had provided a “window of opportunity” to study the mental health of healthcare professionals in the aftermath of such violence.

### Workplace violence and mental health

Workplace violence has become an international public health issue in the past decade (Zhao et al., 2016; Tian et al., 2020). Healthcare professionals are one of the groups most vulnerable to WPV (Kamchuchat et al., 2008; Tian et al., 2020). Across the literature related to WPV occurred in hospitals, the types of WPV contains physical, verbal, emotional or psychological, and sexual violence (Njaka et al., 2020). WPV can cause traumatic influence on healthcare professionals, including physical damage (Coles et al., 2007), feelings of fear and depression (Kobayashi et al., 2020), anxiety (Zhao et al., 2018), sense of insecurity (Kobayashi et al., 2020), suicide and Post-Traumatic Stress Disorder (PTSD) (Koritsas et al., 2007), sense of testiness and helplessness (Chapman et al., 2009), and other mental issues. Apart from the direct impact, the indirect effect of the WPV issue can be amplified through media, social networks, the internet, and then exert negative impact on healthcare professionals (Yang, 2016; Zhao et al., 2018). Based on the abovementioned literature, we propose the following:

Hypothesis 1: Healthcare-related WPV issues (issue salience) can enhance healthcare professionals’ psychological burden.

### Risk perception as a mediator

Given the focus on the association between issue salience and mental health status, we further investigated how issue salience is associated with mental health status. One plausible mediator is risk perception, which is highlighted in risk-related studies (Rogers et al., 2007; Acuña-Rivera et al., 2014; Demuth et al., 2016; Zambrano-Cruz et al., 2018). Risk perception can be seen as a pathway through which issue salience may exert influence on psychological burdens in a risky context (Yang, 2016). Specifically, issue salience can be amplified through media coverage and social networks and enhance individuals’ risk perception (Scherer and Cho, 2003; Humphrey, 2007). Those who were exposed to WPV issues may have incremental risk perception and thus the increased psychological burden (Yang, 2016). The psychological burden may be presented as PTSD (Zafar et al., 2016), anxiety (Zhao et al., 2016), and depression (Hassankhani et al., 2018).

To investigate this hypothetical mediated effect, the association between risk perception and issue salience and the association between risk perception and mental health status should be further discussed, respectively.

The association between issue salience and risk perception was positively correlated in risk-related studies (Yang, 2016). Issue salience was considered as a prominent factor that predicts an individual’s risk perception (Yang, 2016). Specifically, sensational information disseminates through media, social networks and the internet, and thus increases individuals’ risk perception (Kasperson et al., 2013). This is also aligned with a previous study (Brug et al., 2004), which argued that during the SARS outbreak, people received broad media attention with regard to SARS, and individuals’ incremental SARS-related risk perception was observed. In addition to the impact of media and the internet, interpersonal relationships and social networks also function as information sources. A previous study emphasized that issue salience from social networks may also predict risk perception (Yang, 2016). Similarly, among healthcare professionals, those who were highly exposed to WPV-related information can also be affected and have incremental risk perception (Christakis and Fowler, 2013).

Risk perception is the cognitive response and assessment for threats (Han et al., 2021), which stresses its importance as a prominent determinant of individuals’ affective reaction, or even negative mental health symptoms (Sjöberg, 2000). For example, Qing Han and his colleagues focused on risk perception of COVID-19 and argued that risk perception was inversely associated with subsequent mental health (Han et al., 2021). According to social stress theory and related empirical evidence (Yang and Chu, 2018), higher risk perception had a significant association with higher levels of negative emotions, such as feeling lonely, bored, anxious, etc. Meanwhile, risk perception functioned as a risky predictor for the levels of positive emotions, like relaxed, calm, happy. In addition, the findings of Ding et al. (2020) also suggested that risk perception was positively associated with depression symptoms during the COVID-19 outbreak. Thus, based on the associations between issue salience, mental health status, and risk perception, we propose the second hypothesis as follow:

Hypothesis 2: Risk perception functions as a mediator between issue salience and mental health symptoms.

To test these hypotheses, our research conducted a cross-sectional survey among healthcare professionals in China after Dr. Yang Wen was brutally murdered by a relative of a patient.

## Materials and methods

### Sampling strategy and participant details

A cross-sectional study was conducted from January 16, 2020 to February 6, 2020 to explore the psychological status of healthcare professionals. A multi-stage random cluster sampling method was used to select target samples. The survey was in the form of questionnaires and was distributed 5 provincial AAA level hospitals or affiliated hospitals of top universities (also AAA level) in China. Further, one department of selected hospital was sampled randomly, and every healthcare professional therein was asked to complete the questionnaire, including doctors, nurses, nutritionists and administrative staffs. Participation was voluntary and written informed consent was received before the respondents began the questionnaires. The study was approved by the School of Social Development and Public Policy of Beijing Normal University.

In total, 997 healthcare professionals completed the questionnaires. Before data processing, 32 questionnaires completed after January 25, 2020 were discarded since those respondents had completed after the outbreak of COVID-19 in Wuhan and those questionnaires may bias healthcare professionals’ risk perception. Ultimately, we collected 965 valid samples for further analyses.

### Measurements

#### Issue salience

According to prior study, healthcare professionals’ awareness of the WPV incident was used to measure issue salience (Yang, 2016). Hence, we asked participants regarding the awareness of such WPV incident by using the question “To what extent do you know about the incident that happened to Dr. Yang Wen?” Participants were required to answer the question using a 5-point Likert scale.

#### Risk perception

Wilson and his colleagues conducted a study to investigate the accurate measurement of risk perception, and they argued that the measurement that consists of probability and consequence was more accurate than simply asking “How risky is X?” (Wilson et al., 2019). Hence, we asked participants “What is the probability for you to encounter such an event in your place?” and “To what extent does such incident affect you negatively?” Both questions were answered using a 5-point Likert scale.

#### Mental health status

The mental health status of healthcare professionals was measured with three scales: the Checklist-Civilian Version (PCL-C) for measuring PTSD, the Self-rating Depression Scale (SDS) for measuring depression and the Self-rating Anxiety Scale (SAS) for measuring anxiety. The constructs of the three scales are as follows:

#### Post-traumatic stress disorder symptoms

Post-traumatic stress disorder was assessed by the PTSD Checklist-Civilian Version (PCLC). The scale was initially developed by The National Center for Post-Traumatic Stress Disorder (NCPTSD) in 1994. Then, the PCLC was modified and extensively used as a self-report instrument in China (Li et al., 2010; Zhang et al., 2012). The Chinese version of the PCLC includes 17 items (Li et al., 2010; Zhang et al., 2012), and its internal consistency for assessing healthcare professionals’ PTSD performs well (Tang et al., 2017; Zhou et al., 2018). In our research, the PCLC was employed to investigate the PTSD symptoms of healthcare professionals in the aftermath of “death of Dr. Yang Wen.” The participants were asked to answer 17 questions using a 5-point Likert scale. The internal consistency (Cronbach’s alpha) of the PCLC for healthcare professionals was 0.96, showing an acceptable result for this instrument. The total score equaled the sum of all items and ranged from 17 to 85. Probable PTSD symptoms was considered when the healthcare professional had a total PCLC score 38 or more than 38 (Ning et al., 2017; Song et al., 2021).

#### Depression symptoms

The scale for assessing depression symptoms originated from W.K. Zung’s Self-rating Depression Scale (SDS), designed in 1965 (Zung, 1965; Rodriguez-Morales et al., 2018). The Chinese version of the SDS was modified to show acceptable internal consistency and had been extensively used in the researches with regard to healthcare professionals (Chen et al., 2020; Xing et al., 2021). The SDS consists of 20 items, and its internal consistency coefficient (Cronbach’s alpha) was 0.86. The participants were asked to answer each question using a 4-point response option (1 = not at all, 2 = seldom, 3 = often, 4 = very often). The total sum score was then multiplied by 1.25 and consequently ranged from 25 to 100 (Chen et al., 2021). The cutoff point was set to 53, indicating that those who score 53 or more than 53 may have at least moderate depression (Xing et al., 2021).

#### Anxiety symptoms

The scale for assessing anxiety in our research was the Self-rating Anxiety Scale (SAS). Similar to W.K. Zung’s Self-rating Depression Scale (SDS), the SAS has also been extensively used in China (Li et al., 2020; Xing et al., 2021). The sum score of the SAS was also multiplied by 1.25. The scale consists of 20 items, and its internal consistency coefficient (Cronbach’s alpha) was 0.87. The participants were asked to answer each question using a 4-point response option (1 = not at all, 2 = seldom, 3 = often, 4 = very often). The cutoff point was set to 50, SAS total scores 50 or greater than 50 suggested the symptoms of anxiety (Zung, 1971; Dunstan and Scott, 2020).

#### Potential confounding variables

Multiple demographic variables were included in the research, such as gender, types of occupation (doctor, nurses, nutritionists, and administrative staff), marital status, and income. Since self-reported health status and recent traumatic experience can be associated with psychological health status, those variables were also included (Sawatzky et al., 2010; Liu et al., 2017).

### Statistical analyses

We used STATA for the statistical analysis, which entailed descriptive analysis, bivariate analysis, linear regression analysis, curvilinear regression analysis, and mediation analysis. Descriptive analysis was used for the summary of all variables. Bivariate analysis and linear regression were conducted to examine the relationship between issue salience and mental health status. Further, the curvilinear effect was tested by including issue salience as a main effect (linear term), along with a squared (quadratic) term to examine the potential for curvilinear associations. In addition, the mediation analysis was conducted to explore the mediating effect of risk perception on the relationship between issue salience and mental health.

## Results

### Descriptive analyses

The demographic characteristics of the participants were shown in Table 1. Of the 965 respondents who met our criteria, 74.20% were female, 71.50% were married, and 94.40% were without religious beliefs. In terms of types of occupation, the majority of the participants were doctors (48.70%) and nurses (43.73%), while the proportions of nutritionists and administrative staff were 6.01% and 1.55%, respectively. On average, the participants had at least 10 years of work experience. Furthermore, the answers to income satisfaction were measured by a 5-point Likert scale from very dissatisfied to very satisfied, and only 6.94% of participants were reported to have a very dissatisfied attitude toward their income. Moreover, approximately 11% of the respondents reported that they were exposed to traumatic events.

**TABLE 1:** Demographic characteristics of healthcare professionals in China (*N* = 965).

Variables	Frequency	Percent (%)
**Types of occupation**		
Doctor	470	48.70
Nurse	422	43.73
Nutritionist	58	6.01
Administrative staff	15	1.56
**Gender**		
Male	249	25.80
Female	716	74.20
**Marital status**		
Married	690	71.50
Unmarried	261	27.05
Widowed and divorced	14	1.45
**Religion**		
Not have	911	94.40
Have	54	5.60
**The average income per month (CNY)**		
<5,000	98	10.16
5,000–10,000	525	54.40
10,000–20,000	292	30.26
20,000–30,000	39	4.04
>30,000	11	1.14
**Satisfaction with income**		
Very dissatisfied	67	6.94
Partially dissatisfied	170	17.62
General	467	48.39
Partially satisfied	231	23.94
Very satisfied	30	3.11
**Issue salience**		
Not at all	5	0.52
Less	51	5.28
Moderate	267	27.67
More	546	56.58
Extremely	96	9.95
**The probability of violence**		
Not at all	21	2.17
Less	479	49.64
Moderate	161	16.68
More	184	19.07
Extremely	120	12.44
**Emotional impact**		
Not at all	62	6.43
Slight	313	32.44
Moderate	197	20.41
Severe	288	29.84
Extremely severe	105	10.88
**Self-reported health status**		
Very bad	22	2.28
Partly bad	172	17.82
General	481	49.85
Partially good	249	25.80
Very good	41	4.25
**Traumatic experience**		
Have	103	10.67
Not have	862	89.33
**PTSD symptoms (Cutoff points = 38)**		
No	718	74.40
Yes	247	25.60
**Depression symptoms (Cutoff Point = 50)**		
No	521	53.99
Yes	444	46.01
**Anxiety symptoms (Cutoff point = 50)**		
No	696	72.12
Yes	269	27.88
**Variables**	**Mean**	**Standard deviation**
Years of working	10.22	8.05

Regarding the WPV issue, the rates from “Not at all” to “Extremely” were 0.52, 5.28, 27.67, 56.58 and 9.95%, respectively. Only 6.42% of the health care workers reported that they had not been emotionally affected by the WPV issue. More than 25% of healthcare professionals suffered from PTSD symptoms in our study. Meanwhile, the prevalence rates were 46.01% with depression symptoms and 27.88% with anxiety symptoms. In addition, only 2.18% of healthcare professionals believed they had zero possibility of suffering from WPV issues. More details were listed in Table 1.

### Linear regression analyses

Table 2 displayed the results of linear regression analyses of the factors associated with mental health status, including symptoms of PTSD, depression and anxiety. The association between PTSD symptoms and the awareness of healthcare professionals regarding the WPV-incident was significant (coefficient = 1.45, *P* = 0.003). Furthermore, mental health status was significantly related to the type of occupation, religion, marital status, traumatic experience, income and self-reported health status. In detail, compared with doctors, nurses were more likely to report higher anxiety symptoms (coefficient = 2.05, *P* = 0.014), nutritionists were found to have much lower PTSD scores (coefficient = −4.06, *P* = 0.014) and depression symptom scores (coefficient = −3.51, *P* = 0.031), and administrative staff reported much higher depression symptom scores (coefficient = 6.52, *P* = 0.024). Overall, health professionals faced varying degrees of mental health stress. In comparison, those with religious beliefs were more likely to report more symptoms of PTSD (coefficient = 3.48, *P* = 0.026), depression (coefficient = 4.20, *P* = 0.006), and anxiety (coefficient = 4.41, *P* = 0.002). Marital status and traumatic experience were both found to be significantly associated with mental status. Individuals without spouses tended to have somewhat lower PTSD (coefficient = −2.39, *P* = 0.010), and those without traumatic experience were more likely to have considerably better mental health status, including PTSD (coefficient = −5.81, *P* = 0.000), depression (coefficient = −2.45, *P* = 0.037) and anxiety (coefficient = −4.03, *P* = 0.000). The prevalence of PTSD symptoms was significantly higher among the participants with higher income (coefficient = 1.15, *P* = 0.043), whereas no significant relationship was found between income and depression/anxiety. Meanwhile, the level of satisfaction with income was significantly related to individual’s mental health status. Those who were more satisfied with their income tended to have considerably fewer mental health issues, including PTSD (coefficient = −2.68, *P* = 0.000), depression (coefficient = −1.90, *P* = 0.000) and anxiety (coefficient = −2.14, *P* = 0.000). Moreover, compared with poor self-reported health status, the participants who reported greater self-reported health status were less likely to be at risk of PTSD (coefficient = −5.12, *P* = 0.000), depression (coefficient = −3.88, *P* = 0.000) and anxiety (coefficient = −4.58, *P* = 0.000). The details were provided in Table 2.

**TABLE 2:** Regression analysis (*N* = 965).

Variables	Univariate regression coefficient (95% CI)			Multivariate regression coefficient (95% CI)		
	PTSD	Depression	Anxiety	PTSD	Depression	Anxiety
Issue salience	1.45 (0.37, 2.52)**	−0.42 (−1.43, 0.59)	−0.23 (−1.18, 0.72)	1.45 (0.48, 2.41)**	−0.14 (−1.10, 0.81)	−0.05 (−0.92, 0.82)
**Occupation** **(ref: doctors)**						
Nurses	−1.73 (−3.37, −0.09)*	0.16 (−1.38, 1.69)	0.40 (−1.06, 1.86)	1.46 (−0.36, 3.29)	1.45 (−0.34, 3.25)	2.05 (0.42, 3.69)*
Nutritionists	−7.84 (−11.25, −4.43)***	−5.56 (−8.75, −2.38)***	−3.94 (−6.96, −9.22)*	−4.06 (−7.28, −0.83)*	−3.51 (−6.70, −0.33)*	−1.39 (−4.30, 1.51)
Administrative staff	−4.17 (−10.59, 2.26)	5.50 (−0.50, 11.51)	−0.36 (−6.06, 5.33)	−2.22 (−7.96, 3.52)	6.52 (0.86, 12.18)*	0.72 (−4.43, 5.87)
Years of working	0.12 (0.02, 0.22)*	0.15 (0.06, 0.24)***	0.10 (0.01, 0.18)*	−0.03 (−0.13, 0.07)	0.09 (−0.00, 0.19)	−0.00 (−0.09, 0.09)
Gender (ref: male)						
Female	−2.05 (−3.87, −0.24)*	0.33 (−1.36, 2.03)	0.59 (−1.01, 2.19)	−1.57 (−3.52, 0.38)	0.10 (−1.82, 2.02)	0.05 (−1.70, 1.80)
**Marital status** **(ref: married)**						
Unmarried	−3.51 (−5.29, −1.72)***	−2.01 (−3.68, −0.34)*	−2.14 (−3.72, −0.56)**	−2.39 (−4.21, −0.57)**	−0.78 (−2.58, 1.01)	−1.37 (−3.01, 0.27)
Widowed and divorced	3.01 (−3.62, 9.64)	1.81 (−4.40, 8.03)	3.98 (−1.87, 9.83)	3.34 (−2.57, 9.26)	1.29 (−4.55, 7.12)	4.13 (−1.18, 9.44)
**Religion (ref: no)**						
Yes	2.58 (−0.88, 6.05)	3.44 (0.21, 6.66)*	3.77 (0.73, 6.81)*	3.48 (0.42, 6.54)*	4.20 (1.18, 7.22)**	4.41 (1.66, 7.16)**
Income	1.29 (0.24, 2.35)*	0.33 (−0.66, 1.31)	0.46 (−0.47, 1.39)	1.15 (0.03, 2.26)*	0.29 (−0.80, 1.39)	0.78 (−0.22, 1.77)
Satisfaction with income	−2.93 (−3.79, −2.07)***	−2.23 (−3.04, −1.43)***	−2.44 (−3.20, −1.68)***	−2.68 (−3.53, −1.82)***	−1.90 (−2.74, −1.05)***	−2.14 (−2.91, −1.37)***
Self-reported health status	−6.07 (−6.95, −5.19)***	−4.56 (−5.41, −3.71)***	−5.26 (−6.04, −4.48)***	−5.12 (−6.01, −4.23)***	−3.88 (−4.75, −3.00)***	−4.58 (−5.38, −3.79)***
**Traumatic experience** **(ref: yes)**						
No	−7.62 (−10.16, −5.09)***	−3.40 (−5.79, −1.00)**	−5.06 (−7.31, −2.82)***	−5.81 (−8.14, −3.48)***	−2.45 (−4.74, −0.15)*	−4.03 (−6.12, −1.94)***

*P < 0.05, **P < 0.01, ***P < 0.001.

### Curvilinear regression analyses

Taking the TMGT effect (the Too Much of a Good Thing Effect) into account (Pierce and Aguinis, 2013), our research entered issue salience (the linear term) and squared issue salience (the squared term) as predictors to fit the curve association between issue salience and mental health status. The results (presented in Table 3) explained that the squared terms for PTSD (coefficient = 1.80, *P* = 0.000) and anxiety (coefficient = 0.98, *P* = 0.022) were both positive and significant. Furthermore, the linear term was also significantly associated with PTSD (coefficient = −11.49, *P* = 0.001) and anxiety symptoms (coefficient = −7.22, *P* = 0.017), which suggested that higher awareness of the issue was negatively related to mental health status (including PTSD and anxiety) but only to the points at which there were no additional effects of more issue salience. However, issue salience and its squared term were insignificantly associated with depression symptoms, which was in line with the results of the linear regression analysis presented in Table 2. In addition, sociodemographic, traumatic experience and other potential confounding variables had been controlled in the models.

**TABLE 3:** Curve fitting with linear regression (*N* = 965).

Variables	PTSD	Depression	Anxiety
Issue salience	−11.49 (−18.09, −4.89)***	−4.33 (−10.63, 1.98)	−7.22 (−13.14, −1.29)*
Squared Term of issue salience	1.80 (0.87, 2.74)***	0.55 (−0.34, 1.45)	0.98 (0.14, 1.82)*

*P < 0.05, ***P < 0.001.

### Examination of mediating effect

**FIGURE 1:**
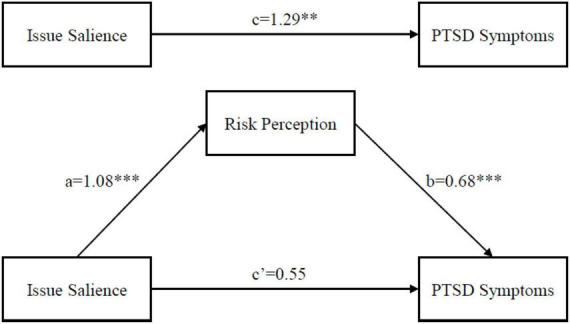
Model of risk perception as a mediator between issue salience and PTSD symptoms. **P < 0.01, ***P < 0.001.

To test Hypothesis 2, mediation analysis was used to assess the associations between issue salience, risk perception, and PTSD symptoms. After controlling for sociodemographic, traumatic experience, self-reported health status and other potential confounding variables, the mediating effect analysis was conducted, and the results were presented in Figure 1. Consistent with the linear regression model, issue salience was also associated with symptoms of PTSD in the total effect model (coefficient = 1.29, *P* = 0.010). The direct effect of issue salience on PTSD symptoms was 0.55; however, the coefficient was insignificant. Furthermore, the direct effect of issue salience on risk perception was 1.08 (*P* = 0.000), and the direct effect of risk perception on the symptoms of PTSD was 0.68 (*P* = 0.000). Moreover, the path a, path b and path c were positive, indicating that risk perception played a mediating role between issue salience and PTSD symptoms. To summarize, the results confirmed that risk perception completely mediates the relationship between risk information and PTSD since the effect of risk information on PTSD became insignificant when risk perception was included; thus, Hypothesis 2 was supported. Therefore, risk perception was a strong predictor and mediator. The details can be seen in Figure 1.

## Discussion

A serious of studies tracing back to the intricate factors of medical disputes and WPV against healthcare personnel have been providing, includes health system reform, government factors, along with medical service institutions factors (Wu and Trigo, 2020). Consequently, those factors had increased the doctor-patient conflict to a certain extent (Duan et al., 2014). Thus, WPV against healthcare personnel such as doctor, nurse, nutritionist, and administrative staff has been accumulating generally. Aside from direct impact from WPV, the indirect impact should also be emphasized. Therefore, we aimed to discuss the association between issue salience and mental health status, and mediation effect of risk perception on the relationship between issue salience and mental health.

### Association between issue salience and mental health status

Linear regression analysis and curvilinear regression analysis were conducted to examine the relationship between issue salience and mental health status. In our research, linear regression analyses showed that issue salience was positively associated with PTSD symptoms, whereas the relationship between issue salience and depression symptoms and the relationship between issue salience and anxiety symptoms remained insignificant. One possible explanation for the association between issue salience and PTSD symptoms, is that each WPV issue is a potential traumatic incident that causes individual emotional exhaustion and thus leads to PTSD symptoms (Maguen et al., 2009). Apart from direct exposure to WPV, it was thought that indirect trauma, such as exposed to WPV information, can be a trigger for PTSD symptoms (Szogi and Sullivan, 2018). Similar to other individuals threatened by WPV issues, healthcare professionals tended to acquire WPV-related information through social networks and media, internalize the information into empathetic emotions, and generate cognitive and emotional alternatives, namely, feeling as if they were the victim themselves (Regehr et al., 2002). The empathy emotion process would fail to emotionally differentiate healthcare professionals themselves and real WPV-related victims (Badger et al., 2008) and could eventually lead to PTSD symptoms. With regard to issue salience, depression and anxiety, WPV issue salience was insignificantly associated with depression and anxiety. In contrast, previous studies argued that WPV had a significant impact on individuals’ depression and anxiety (Paterson et al., 2010; Zhao et al., 2018). One possible assumption for the discrepancy is that such insignificance can be attributed to the method of WPV-related information dissemination. Participants in our research suffered indirect trauma through social networks, extensive media coverage and news reports on the internet, whereas participants in other studies may experience WPV issues in person (Zhao et al., 2018). In sum, WPV issue salience was associated with PTSD but was insignificantly correlated with depression and anxiety. However, the insignificance may be due to the too-much-of-a-good-thing effect (TMGT effect); thus, curvilinear regression analysis is required.

Curvilinear regression was conducted to investigate the U-shaped relationship between issue salience and PTSD symptoms, depression and anxiety, respectively. First, the results indicated that issue salience changed from low to moderate and reduced an individual’s PTSD symptoms only to a tipping point. Beyond this tipping point, when issue salience elevated, PTSD can also increase from moderate to high. This can be explained by the fact that issue salience may trigger an individual’s fear denial mechanism (Aronson, 2008) and may serve as an anesthetic in that individuals may be too shocked to accept the WPV-related issue. Then, beyond the tipping point, as individuals learned more about the details of the murderer’s inhumanity in the event, they tended to exhibit more PTSD symptoms. Moreover, the results of anxiety in curvilinear regression suggested that the association between issue salience and anxiety was significant, indicating that issue salience may reduce healthcare professionals’ anxiety level before the tipping point and then may elevate anxiety levels beyond the tipping point. This quadratic relationship can be explained by the fact that increasing awareness (information from multiple sources) may lead to an individual’s indifference, and beyond the tipping point, individuals were more likely to suffer from symptom of anxiety when they perceived more information concerning detailed WPV-related issues. Unlike PTSD and anxiety, depression showed an insignificant relationship with issue salience. The inconsistency between these findings could be explained for the following reasons. Specifically, PTSD and depression (Elhai et al., 2008) or anxiety and depression (Ramakrishna et al., 2019) often occur as comorbid illness following traumatic events, individuals with depression symptoms were more likely to be diagnosed with concurrent PTSD or anxiety symptoms (Breslau et al., 2000; Falade et al., 2020). In addition, previous studies suggested that PTSD symptoms functioned as a predictive factor for the development of depression (Walecka et al., 2021). PTSD could affect individuals’ normal emotional processing (Walecka et al., 2021), thereby exacerbating negative thoughts about the world. The emotional process is congruent with the characteristic of depression according to Beck’s cognitive theory of depression (Xu and Zhang, 2016). Anxiety, fear and denial caused by PTSD could also encourage individuals to withdraw from interpersonal relationship (Wei et al., 2005), which can further lead to or aggravate the occurrence of depression (Wei et al., 2005). Similarly, anxiety and depression were highly correlated (Wetherell et al., 2001), anxiety was not only a risk predictor of depression disorder, but considered to be the prodromal stage of depression (Wittchen et al., 2003). Thus, issue salience may predict depression though PTSD or anxiety, that is why issue salience did not relate to depression in the present study.

### The mediation effect of risk perception

Our research examined whether risk perception mediated the relationship between issue salience and the symptoms of PTSD and found a significant mediation effect. Specifically, it is noteworthy that issue salience was positively correlated with risk perception, which is consistent with prior studies highlighting increased risk perception among individuals in the aftermath of risk events (Van Der Pligt, 1985; Yang, 2016). For example, risk perception can be strongly affected by awareness of nuclear energy due to the potential risk of reactor operation and the possible health hazards associated with nuclear energy (Van Der Pligt, 1985). Similarly, exposure to information highlighting the impact of WPV issues elevated healthcare professionals’ risk perception. Furthermore, the relationship between risk perception and PTSD symptoms embedded in the mediation model was aligned with the previous study (Wu et al., 2009). It was reported that risk perception could affect individuals’ likelihood of PTSD because of the unfamiliarity and perceived uncontrollability of hazards (Wu et al., 2009). Similar results had also been reported in the research in context of COVID-19 (Han et al., 2021). Risk perception may exert effects on their psychological wellbeing through weakening their positive emotion or stimulating negative emotions. The phenomenon could also be explained by the Lazarus and Folkman theoretical approach, which indicated that risk perception as one form of primary appraisal of a threat situation could determine the stress response (Brug et al., 2004). To summarize, risk perception mediated the effect between issue salience and psychological wellbeing. Moreover, issue salience was not significantly correlated with PTSD symptoms after entering risk perception as a mediator. Individuals who have paid more attention to WPV issues were more likely to show PTSD symptoms rather than issue salience affecting PTSD symptoms directly. Thus, it may be important to intervene risk perception to achieve the symptoms of PTSD reduction.

Moreover, our research also involved other variables with theoretical relevance for mental health status, such as income, satisfaction with income, gender, marital status, traumatic experience, self-reported health status, religion and years of working. First, the results indicated that income was a risk factor for developing PTSD symptoms. This result appears to contrast with Patel (2007), who found that income was an important protective predictor for mental health status since individuals with higher income were more likely to have sufficient mental health care and services. Moreover, satisfaction with income was strongly associated with mental health status, including PTSD, depression and anxiety. Specifically, responders with a high level of income satisfaction reported better mental health status. The inconsistent results on income and income satisfaction on mental health could be explained by the fact that the perception of real purchasing power can be more influential than that of actual income. Second, there was no significant difference between gender and mental health status, including PTSD, depression and anxiety symptoms, which is in line with a previous study conducted among healthcare professionals in China (Liu Y. et al., 2021). With regard to marital status differences, our results suggested that a higher level of mental health issues was presented in individuals with spouses, since they were also the providers of childcare and housework and thus were more tired and stressed (Chen et al., 2010). In addition, as reported in the literature conducted among healthcare professionals (Greenberg et al., 2020), individuals with other traumatic experiences were more likely to develop mental health issues in our research. With regard to self-reported health status, better self-reported health status tended to predict normal mental health status, which is congruent with a prior study (Andrew and Dulin, 2007). Moreover, religious belief was a risk factor for mental health in our research, whereas Schmuck and his colleagues found that religion provided individuals with spiritual support and thus played a protective role (Schmuck et al., 2021). In addition, the results indicated no relationship between years of working and mental health status. Based on our research results, prevention efforts for healthcare professionals need to focus more on those who are at greater risk of mental health issues.

## Conclusion

The relationship between issue salience and healthcare professionals’ PTSD symptoms had been confirmed in our research. Further, risk perception was shown to mediate the effect of WPV awareness on PTSD symptoms. In addition, our study noticed a U-shaped relationship between issue salience and PTSD, and U-shaped relationship between issue salience and anxiety as well. Accordingly, the underestimated WPV issue should be brought to attention, and thus more protective measures for healthcare professionals need to be implemented in response to WPV events:

1. Governments and hospitals should pay more attention on training programs in order to improve healthcare professionals’ understanding of violence and coping skills.
2. In the aftermath of WPV issues, timely psychological interventions and social support should be provided for healthcare professionals. Since risk perception was found to mediate the effect of WPV issue salience on PTSD symptoms, it is critical to diminish the mental health burden of healthcare professionals through intervening in risk perception.

However, there were also limitations in our research. The research was limited by a cross-sectional design, the results cannot be used to draw causal inference conclusions. Additionally, although we have considered risk perception and sociodemographic variables, the mental health status of healthcare professionals is a complex system, and we cannot enumerate every relevant variable. Besides, in order to interpret the health care worker’s experience from the story such as “the attack on Dr. Yang Wen,” further study needed to be conducted by qualitative research.

## Data availability statement

The original contributions presented in the study are included in the article/supplementary material, further inquiries can be directed to the corresponding author/s.

## Ethics statement

The studies involving human participants were reviewed and approved by the School of Social Development and Public Policy of Beijing Normal University. Participants provided written informed consent to participate in the study.

## Author contributions

QZ, DZ, and CCL contributed to the conceptualization, methodology, data curation, formal analysis, writing—original draft, and editing. HS contributed to the conceptualization, investigation, and writing—review and editing. CNL contributed to investigation, funding acquisition, and project administration. WL and JC contributed to investigation. All authors contributed to the article and approved the submitted version.
